# Association of Sports Practice in Childhood and Adolescence with Cardiac Autonomic Modulation in Adulthood: A Retrospective Epidemiological Study

**DOI:** 10.1186/s40798-024-00707-7

**Published:** 2024-04-16

**Authors:** Diego Giulliano Destro Christofaro, William R. Tebar, Jorge Mota, Leandro D. Delfino, Amanda B. Santos, Raphael M. Ritti-Dias, Rômulo A. Fernandes, Gerson Ferrari, Luiz Carlos M. Vanderlei

**Affiliations:** 1 School of Technology and Sciences, São Paulo State University (Unesp), Roberto Simonsen Street, n° 305, Presidente Prudente, São Paulo, 19060-900 Brazil; 2 Centre of Clinical and Epidemiological Research, University Hospital, University of Sao Paulo, São Paulo, Brazil; 3 Research Center in Physical Activity, Health and Leisure (CIAFEL), Faculty of Sports, University of Porto (FADEUP) and Laboratory for Integrative and Translational Research in Population Health (ITR), Porto, Portugal; 4 Graduate Program in Rehabilitation Sciences, Universidade Nove de Julho - UNINOVE, São Paulo, Brazil; 5 Faculty of Health Sciences, Universidad Autónoma de Chile, Providencia, Chile

**Keywords:** Heart rate variability, Previous sports practice, Youth, Cardiovascular Health

## Abstract

**Background:**

Practicing sports during childhood and adolescence provides benefits to cardiac autonomic modulation (CAM) at these stages of life. However, it is not known whether these benefits to CAM persist into adulthood. Therefore, the objective of this study was to analyze the association of early sports practice (sports practice in childhood and/or adolescence) with CAM in adult life, regardless of habitual moderate-to-vigorous PA.

**Methods:**

The sample of the present study consisted of 242 adults (141 women and 101 men; age: 41.99 ± 16.24). The assessment of CAM was performed using heart rate variability indices. Sports practice in childhood and adolescence was assessed using a questionnaire. The intensity of physical activity was assessed using accelerometry. To analyze the association between previous sports practice (childhood and/or adolescence) and CAM, the Generalized Linear Model was adopted, considering CAM indices as continuous variables and early sports practice as a 3-fold factor (no sports practice; sports practice in childhood or adolescence; and sports practice in both childhood and adolescence) adjusted by sex, age, socioeconomic condition, and moderate to vigorous PA.

**Results:**

Sports practice in childhood was associated with the average standard deviation of all normal RR intervals expressed in milliseconds (SDNN): β = 5.89; 95%CI: 0.25;11.52, and the standard deviation of the long-term intervals between consecutive heartbeats (SD2): β = 7.63; 95%CI:1.04; 14.23 indices. Sports practice in adolescence was associated in adulthood with the SD2 index: β = 7.37; 95%CI: 0.71;14.04. Sports practice in at least one of the periods (childhood or adolescence) was significantly associated with the square root of the mean square of the differences between adjacent normal RR intervals for a period of time expressed in milliseconds (RMSSD) (β = 8.86; 95%CI = 0.71;17.01), and the standard deviation of the instantaneous beat to beat variability (SD1) (β = 6.21; 95%CI = 0.45;11.97). Sports practice at both stages of life was significantly associated with better SDNN (β = 7.70; 95%CI = 1.16;14.23) and SD2 (β = 10.18; 95%CI = 2.51;17.85).

**Conclusion:**

Early sports practice was associated with better CAM in adulthood, independently of the current physical activity level. Based on these findings, sports practice is encouraged from childhood and adolescence, for benefits to CAM in adult life.

## Key Points

Few studies have verified whether the practice of sports in childhood and adolescence could be associated with better CAM in adult life.

Sports practice in childhood and/or in adolescence was associated with better CAM in adult life, both when analyzed separately and when combined.

Early sports practice was associated with indices of cardiac autonomic modulation in adulthood that reflect global variability (SNDD and SD2) and parasympathetic modulation (RMSSD and SD1).

## Background

Cardiac autonomic modulation (CAM) is an important cardiovascular risk marker, and low CAM values have been associated with higher odds of mortality. Koopman et al. [1] in a study with 822 adults (median age 65 years) observed that low global CAM was associated with higher mortality risks. Sen and McGill [2], in a systematic review with meta-analysis addressing prospective studies, also showed that low CAM can be an important predictor of mortality.

In this sense, lifestyle habits that can contribute to increases in CAM, and consequently decrease the chances of mortality, should be encouraged, including the practice of physical activity (PA). Christofaro et al. [3] observed that moderate and vigorous PA were associated with greater global and parasympathetic CAM in men, while light PA was associated with lower sympathetic activity in women. Corroborating these findings, Pope et al. [4] in a study with American adults observed that the practice of PA was associated with better CAM. These relationships have also been investigated in athletes and children [5, 6].

However, despite the important findings mentioned above, the effects of prior PA practice performed in childhood and adolescence on the health of the adult population have only recently been investigated. Childhood and adolescence are important phases of life [7, 8], in which lifestyle habits such as the practice of PA were carried into adult life [9, 10]. Silva et al. [11] in a study analyzing the practice of PA performed through sports, observed that adults who practiced this type of PA in childhood and adolescence were less likely to have hypertension in adult life. Similar findings were observed by Fernandes et al. [12] in which adults who practiced sports in childhood and adolescence were less likely to have dyslipidemia in adult life. Werneck et al. [13] found similar findings between previous sports practice and adiposity in adults.

However, after searching the scientific literature, no studies were found that investigated the relationship between previous sports practice and CAM in adult life and whether possible associations between previous sports practice and CAM are independent of habitual PA practice. Furthermore, the present study aimed to control the analysis by directly measuring the participants’ usual physical activity. It is also noteworthy that the majority of studies investigating the relationship between PA and CAM have been carried out in developed countries, with a lack of information from Latin American countries such as Brazil.

Therefore, the objective of the present study was to investigate the association of sports practice in childhood and/or adolescence with CAM in adult life and whether the possible associations observed are independent of habitual moderate-to-vigorous PA.

## Methods

### Sample

The sample of the present study was composed of adults with an average age of approximately 40 years (age:41.99 ± 16.24) from the city of Santo Anastácio, an inner city located in the southeastern region of Brazil. To calculate the sample size, a correlation of *r* = 0.23 between PA and CAM [14], a power of 80%, and an alpha error of 5% were considered, which generated a minimum number of 147 participants. However, anticipating a sample loss due to incomplete completion of the questionnaire, or error in the CAM and PA measures, another 30% were added, which generated a minimum sample number of 191 participants.

**Fig. 1:**
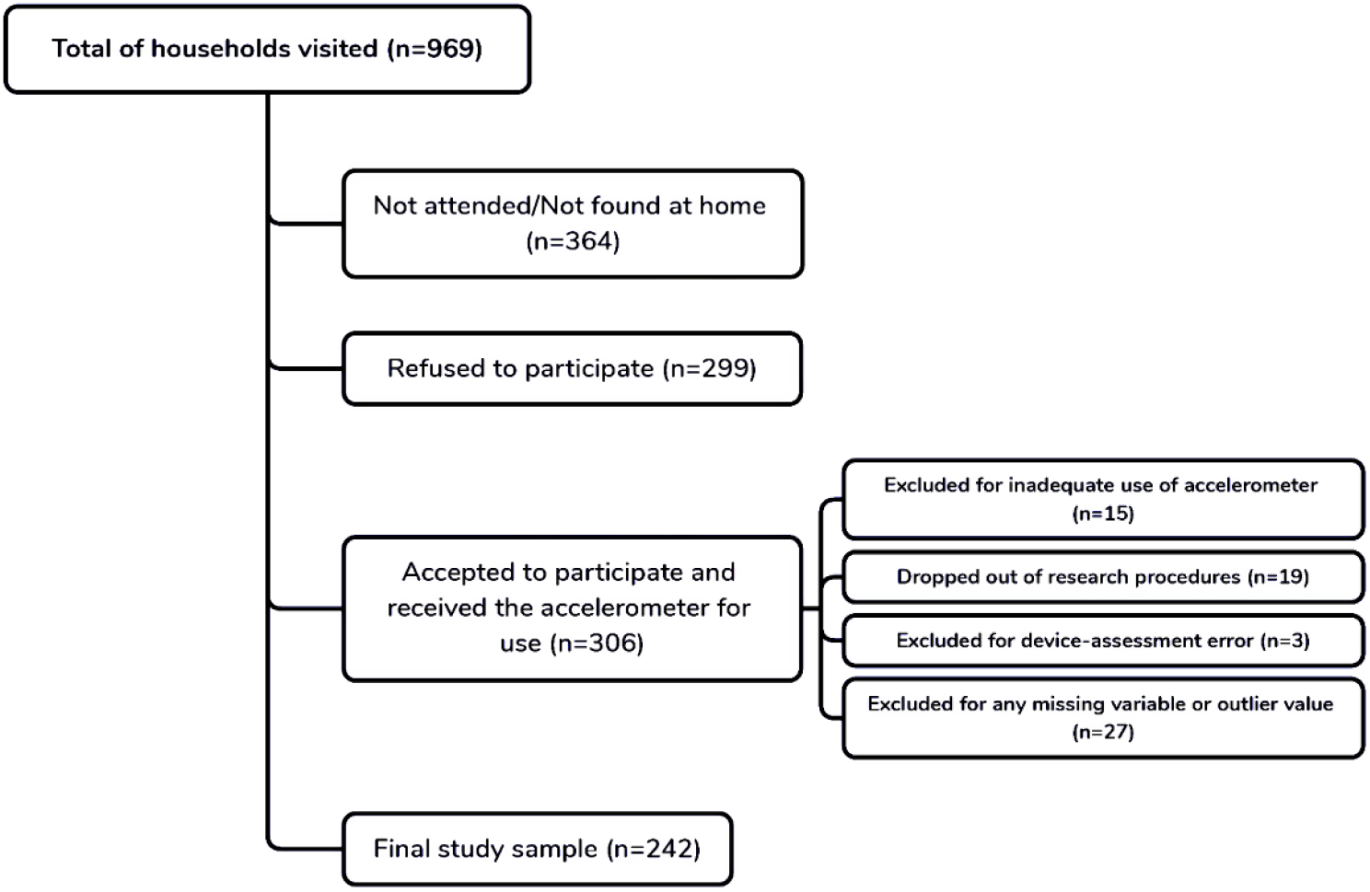
Sampling flowchart

The participants were selected according to the representativeness of each census tract of the city, in a random sampling process stratified by streets, blocks, and households within each census tract. Detailed information on the sampling process is available in the literature [15]. As inclusion criteria, the participants needed to be ≥ 18 years of age; resident in the city for at least 2 years; and sign the informed consent form agreeing to participate in all the research procedures. The exclusion criteria were defined as insufficient use of the accelerometer (less than 10 h for five days); missing part of the research procedure; or having any kind of device-assessment error for the accelerometry or CAM. The complete sampling flowchart is presented in Fig. 1. The study was approved by the Ethics and Research Committee of the São Paulo State University under protocol no. CAAE: 72191717.9.0000.5402 (approved on: August 11, 2017). All participants signed a consent form agreeing to participate in the study. The present study followed all the recommendations of the Declaration of Helsinki. The study was carried out between August 2018 and October 2020.

### Cardiac Autonomic Modulation

The assessment of cardiac autonomic modulation was performed using heart rate variability (HRV). For this evaluation, the subjects were instructed not to consume alcoholic beverages and/or stimulants, such as coffee and tea, for 12 h before the experimental protocol, not to perform physical activity beyond the usual activity for 24 h before the protocol, and to have a good night’s sleep. This was requested to minimize the influences on the cardiac autonomic behavior at the time of the test assessment. To evaluate the CAM using HRV, an electrical impulse capture belt was used, which consists of two electrodes arranged in a sealed electronic transmitter. The strap was positioned on the distal third of the participant’s sternum to capture the beat-to-beat heart rate (RR intervals), at a sampling frequency of 1000 Hz, and information was transmitted through an electromagnetic field to a Polar heart rate receiver, model V800 (Polar Electro Oy, Kempele, Finland) positioned on one of the participant’s wrists.

All participants were evaluated in a room with a controlled temperature (21 to 23ºC) and monitored humidity (40 to 60%). Participants were instructed to remain in silence, awake, at rest in a supine position on a mattress, breathing spontaneously for 30 min, while the heart rate was recorded beat to beat continuously.

The RR intervals were exported from the Polar heart rate receiver and saved in text format by the Polar Flow Web Service (https://flow.polar.com/), before being downloaded and analyzed using Kubios HRV Analysis software, version 2.0 (Kupio University, Finland).

For analysis, the RR interval time series was initially subjected to preprocessing to eliminate premature ectopic beats and artifacts, with a median filter and interval correction, using a cubic spline interpolation method. After preprocessing, the data were visually screened for the absence of artifacts or cardiac arrhythmias that could interfere in the HRV analysis [16, 17]. Only series of RR intervals with more than 95% sinus beats were included in the study. For analysis, the most stable sections of the RR interval series were used, and 1000 consecutive RR intervals were used in the analysis.

For HRV analysis using linear methods in the time domain, the RMSSD index, defined as the square root of the square mean of the differences between adjacent normal RR intervals, in a time interval, expressed in milliseconds (ms), and the SDNN, representing the standard deviation of the mean of all normal RR intervals, also expressed in ms [18] were calculated. For HRV analysis in the frequency domain, low frequency t (LF – 0.04 to 0.15 Hz) and high frequency spectral components (HF – 0.15 to 0.4 Hz), in normalized units, were calculated using a fast Fourier transform algorithm [18].

The indices derived from the quantitative analysis of the Poincaré plot were also obtained. For this analysis an ellipse was fitted to the points of the chart, with the center determined by the average RR intervals, and the standard deviation of the instantaneous beat to beat variability (SD1) indices were calculated to measure the standard deviation of the distances of the points to the diagonal y = x (standard deviation of instantaneous beat-to-beat variability) and standard deviation of the long-term intervals between consecutive heartbeats (SD2), which measures the standard deviation of the distances of points to the line y = - x + RRm, where RRm is the average of RR intervals (long-term standard deviation of continuous R-R intervals) [19]. For analysis of linear and non-linear methods, Kubios HRV Analysis software, version 2.0 (Kupio University, Finland) was used.

### Early Sports Practice in Childhood and Adolescence

To retrospectively evaluate the sports practice in childhood and adolescence, two specific questions were asked: “Did you engage in any organized/supervised sport activities outside of school for at least 1 year during the time when you were 7–10 years old (childhood)?” and “Did you engage in any organized/supervised sport activities outside of school for at least 1 year during the time when you were 11–17 years old (adolescence)?” Possible answers were yes or no [20]. This instrument was adopted as it is easy to understand and highly reproducible (Kappa coefficient = 1.00, *p* = 0.001 for both sports practice in childhood and in adolescence), and has been used previously by several epidemiological studies [21–23].

### Covariates

The variables sex, age, moderate to vigorous physical activity (MVPA), and socioeconomic status were considered as covariates. MVPA was objectively measured using an Actigraph GT3X accelerometer (ActiGraph, LLC, Pensacola, FL, USA) which is a light, portable, easy-to-use instrument designed to be positioned on the subject to record movements in the three orthogonal planes: vertical, horizontal anteroposterior and mediolateral. At recruitment, the participants were personally instructed by research staff about the correct use of the accelerometer, which was required to be positioned laterally on the waistline and used for at least 10 h per day, during five days (being three days on weekdays and two days on weekends) [24]. The participants were also instructed about how to take care of the equipment and that they should wear it throughout the day (waking hours), removing it only for contact with water (personal hygiene or any type of water activity) and while sleeping. The raw data from the accelerometer in counts per minute were joined in 60 s epoch periods (in minutes of activity) and classified as MVPA, according to the cut-off of ≥ 2690 counts recommended by Sasaki et al. [25]. In this sense, the total minutes of activity ≥ 2690 counts during the assessment period were classified as the total weekly minutes of MVPA.

The Brazilian Criteria for Economic Classification were used to assess the socioeconomic status of the sample (ABEP) [26]. This instrument considers the education level and the presence of specific rooms and consumer goods at home, to provide a specific score stratified into economic classes, from the highest to lowest.

### Statistical Analysis

The sample characterization variables are presented as mean and standard deviation. To analyze the association between previous sports practice (childhood and adolescence) and CAM, the Generalized Linear Model was adopted, considering CAM indices as continuous variables and early sports practice as a 3-fold factor (no sports practice; sports practice in childhood or adolescence; and sports practice in both childhood and adolescence). Three multivariate models were created: model 1: unadjusted, model 2: adjusted for sex, age, and socioeconomic status, and model 3: variables from model 2, plus MVPA measured by accelerometry. Statistical significance was set at the *p* < 0.05 level, with a confidence interval of 95%, and analysis was performed using the software IBM SPSS version 25.0.

## Results

The sample of this study consisted of 242 participants, 141 women (58.3%) and 101 men, with an average age of 42.0 ± 16.2. A total of 43.4% of the participants reported having practiced sport in childhood, and 50.4% reported sports practice in adolescence. Only 36% of the sample reported sports practice in both childhood and adolescence. The sample characterization is presented in Table 1.

**Table 1:** Sample characterization variables (*n* = 242)

Variables	Values
Age (Years), mean (SD)	42.0 (16.2)
Women vs. men, n (%)	141 (58.3) vs. 101 (41.7)
Weight (kg), mean (SD)	77.2 (15.9)
Height (cm), mean (SD)	165.8 (9.7)
SDNN, mean (SD)	47.1 (20.1)
RMSSD, mean (SD)	32.4 (24.7)
LF, mean (SD)	63.2 (16.7)
HF, mean (SD)	36.8 (16.7)
SD1, mean (SD)	22.9 (17.4)
SD2, mean (SD)	61.8 (24.8)
MVPA, mean (SD)	12.7 (23.5)
Sports practice in childhood, n (%)	105 (43.4)
Sports practice in adolescence, n (%)	122 (50.4)
Sports practice in both childhood and adolescence, n (%)	87 (36.0)

SD: Standard deviation; SDNN: average standard deviation of all normal RR intervals expressed in milliseconds; RMSSD: square root of the mean square of the differences between adjacent normal RR intervals for a period of time expressed in milliseconds; LF: low frequency component; HF: high-frequency component; and LF/HF: ratio of low frequency component/high-frequency component; SD1 = standard deviation of the instantaneous beat to beat variability; SD2 = standard deviation of the long-term intervals between consecutive heartbeats; MVPA: Moderate to vigorous physical activity

Table 2 presents the association between sports practice in childhood and CAM in adult life. It was observed that sports practice in childhood was associated with SDNN and SD2 indices, regardless of confounding variables, such as sex, age, socioeconomic status, and MVPA. It was noteworthy that MVPA seemed to enhance the association between sports practice in adolescence and these indices when inserted as an adjustment variable in model 3.

**Table 2:** Association between sports practice in childhood and cardiac autonomic modulation in adulthood (*n* = 242)

	Model 1		Model 2		Model 3	
	β(95%CI)	P	β(95%CI)	P	β(95%CI)	P
**SDNN**						
No practice	Reference	---	Reference	---	Reference	---
Practice	9.29 (4.15; 14.32)	< 0.001	5.70 (0.04; 11.38)	0.049	5.89 (0.25; 11.52)	0.041
**RMSSD**						
No practice	Reference	---	Reference	---	Reference	---
Practice	9.21 (2.71; 15.70)	0.005	6.67 (-0.97; 2.92)	0.087	6.93 (-0.72; 14.58)	0.076
**LF**						
No practice	Reference	---	Reference	---	Reference	---
Practice	3.08 (-1.12; 7.29)	0.151	0.96 (-3.55; 5.47)	0.677	0.89 (-3.71; 5.50)	0.704
**HF**						
No practice	Reference	---	Reference	---	Reference	---
Practice	-3.26 (-7.48; 0.95)	0.129	-1.06 (-5.57; 3.45)	0.644	-1.07 (-5.67; 3.53)	0.648
**SD1**						
No practice	Reference	---	Reference	---	Reference	---
Practice	6.32 (1.74; 10.91)	0.007	4.56 (-0.86; 9.99)	0.100	4.76 (-0.67; 10.01)	0.086
**SD2**						
No practice	Reference	---	Reference	---	Reference	---
Practice	12.74 (6.45; 19.02)	< 0.001	7.39 (0.75; 14.04)	0.029	7.63 (1.04; 14.23)	0.023

Model 1: Crude model; Model 2: Model 1 + adjusted by sex, age, and socioeconomic condition; Model 3: Model 2 + MVPA

Sports practice in adolescence was associated with SDNN, RMSSD, SD1, and SD2 in the unadjusted model – Table 3. After inserting the covariates of sex, age, and socioeconomic status, sports practice in adolescence remained associated only with SD2 (*P* = 0.031), while the association with SDNN became marginal (*P* = 0.055). Similar results were observed after the insertion of MVPA as an adjustment variable in model 3.

**Table 3:** Association between sports practice in adolescence and cardiac autonomic modulation in adulthood (*n* = 242)

	Model 1		Model 2		Model 3	
	β(95%CI)	P	β(95%CI)	P	β(95%CI)	P
**SDNN**						
No practice	Reference	---	Reference	---	Reference	---
Practice	9.28 (4.32; 14.16)	< 0.001	5.50 (-0.12; 11.12)	0.055	5.51 (-0.11; 11.11)	0.055
**RMSSD**						
No practice	Reference	---	Reference	---	Reference	---
Practice	8.48 (2.37; 14.59)	0.006	5.37 (-1.75; 15.50)	0.140	5.38 (-1.73; 15.50)	0.139
**LF**						
No practice	Reference	---	Reference	---	Reference	---
Practice	1.87 (-2.32; 6.07)	0.382	0.09 (2.21; -4.26)	0.969	0.08 (-4.26; 4.43)	0.970
**HF**						
No practice	Reference	---	Reference	---	Reference	---
Practice	-2.58 (2.16; -6.77)	0.227	-0.79 (-5.20; 3.61)	0.725	-0.79 (-5.20; 3.62)	0.725
**SD1**						
No practice	Reference	---	Reference	---	Reference	---
Practice	5.81 (1.50; 10.12)	0.008	3.62 (-1.41; 8.67)	0.159	3.63 (-1.40; 8.67)	0.157
**SD2**						
No practice	Reference	---	Reference	---	Reference	---
Practice	12.66 (3.08; 6.61)	< 0.001	7.36 (0.69; 14.04)	0.031	7.37 (0.71; 14.04)	0.030

Model 1: Crude model; Model 2: Model 1 + adjusted by sex, age, and socioeconomic condition; Model 3: Model 2 + MVPA

Table 4 presents the association of the cluster of early sports practice in one or both periods (childhood and adolescence) and CAM. Sports practice in at least one of the periods (childhood or adolescence) was significantly associated with RMSSD and SD1, being marginally associated with SDNN.

**Table 4:** Association between cluster of sports practice in childhood and/or adolescence and cardiac autonomic modulation in adulthood (*n* = 242)

	Model 1		Model 2		Model 3	
	β(95%CI)	P	β(95%CI)	P	β(95%CI)	P
**SDNN**						
No practice	Reference	---	Reference	---	Reference	---
At least one	9.15 (3.52; 14.78)	0.001	6.00 (-0.87; 12.88)	0.087	5.97 (-0.95; 12.87)	0.091
Both	6.68 (0.09; 13.27)	0.047	7.60 (1.03; 14.16)	0.023	7.70 (1.16; 14.23)	0.021
**RMSSD**						
No practice	Reference	---	Reference	---	Reference	---
At least one	8.82 (1.16; 16.48)	0.024	8.93 (0.84; 17.02)	0.031	8.86 (0.71; 17.01)	0.033
Both	7.88 (1.11; 14.65)	0.022	8.12 (-0.63; 18.88)	0.069	8.27 (-0.47; 17.01)	0.064
**LF**						
No practice	Reference	---	Reference	---	Reference	---
At least one	-1.34 (-7.25; 4.57)	0.657	-2.76 (-8.63; 3.10)	0.357	-2.74 (-8.62; 3.13)	0.360
Both	4.00 (-0.57; 8.57)	0.086	0.71 (-4.16; 5.59)	0.773	0.68 (-4.26; 5.62)	0.788
**HF**						
No practice	Reference	---	Reference	---	Reference	---
At least one	1.87 (-4.06; 7.80)	0.537	-1.29 (-6.12; 3.55)	0.602	-1.30 (-6.21; 3.61)	0.603
Both	-4.52 (-9.02; -0.03)	0.049	3.27 (-2.62; 9.17)	0.277	3.28 (-2.62; 9.18)	0.276
**SD1**						
No practice	Reference	---	Reference	---	Reference	---
At least one	6.16 (0.75; 11.56)	0.026	6.27 (0.55; 11.99)	0.031	6.21 (0.45; 11.97)	0.034
Both	5.27 (0.48; 10.05)	0.031	5.52 (-0.68; 11.73)	0.081	5.63 (-0.56; 11.28)	0.075
**SD2**						
No practice	Reference	---	Reference	---	Reference	---
At least one	7.77 (-0.24; 15.78)	0.057	6.40 (-1.84; 14.65)	0.128	6.33 (-1.95; 14.62)	0.134
Both	13.18 (5.99; 20.38)	< 0.001	10.04 (2.31; 17.77)	0.011	10.18 (2.51; 17.85)	0.009

Model 1: Crude model; Model 2: Model 1 + adjusted by sex, age, and socioeconomic condition; Model 3: Model 2 + MVPA

After inserting the confounding covariates of sex, age, socioeconomic status, and MVPA, sports practice in both stages of life was significantly associated with better SDNN and SD2 indices and marginally associated with RMSSD and SD1.

## Discussion

The present study analyzed the associations of early sports practice in childhood and/or adolescence with CAM in adult life. The main findings showed that sports practice in childhood and/or in adolescence was associated with better CAM in adult life, both when analyzed separately and when combined. It was observed that, regardless of the confounding covariates, there was an association between early sports practice and the SDNN and SD2 indices, which indicate global variability, and the RMSSD and SD1 indices, which reflect parasympathetic modulation.

Early sports practice has been related to several health benefits in adulthood, such as lower cardiovascular risk factors [11] and greater physical fitness [27]. In addition, Werneck et al. [13] observed that sports practice in childhood and adolescence was inversely related to body-fat, and subjects who were active their whole lives also had lower levels of C-reactive protein. Fernandes and Zanesco [20], in a study with more than 1400 participants observed that early sports practice was associated with lower rates of arterial hypertension and type 2 diabetes in adult life.

The aforementioned findings corroborate the results observed in the present study of the association between early sports practice with better CAM, which reinforces the importance of early sports practice in childhood and adolescence to reduce risks in adulthood.

It has been observed that lower autonomic modulation in adulthood has been associated with an increased risk of developing cardiovascular diseases. Civejetic et al. [28] observed that CAM was inversely related to adiposity. The underlying mechanism of association between adiposity and CAM can be partially explained by inflammation. Increased inflammation can lead to deregulation of the autonomic nervous system, which would contribute to the reduction in CAM [29]. Haarala et al. [30] analyzing young adults from Finland observed that reduced CAM was independently associated with C-reactive protein, even after adjusting for sex, age, blood pressure, and lifestyle habits. Regarding arterial hypertension, higher blood pressure values were associated with higher sympathetic activity, which would be related to lower CAM [31, 32].

It is important to highlight that early sports practice remained associated with CAM even after adjustment for the current level of moderate-to-vigorous physical activity. This was an important strength of the study, since CAM has been associated with different intensities and domains of physical activity [3, 33]. The results seem to suggest that the benefits of an active lifestyle in youth may also have tracked into further life stages for cardiac autonomic modulation. Nevertheless, the contribution of early sports participation against cardiovascular risk factors in adulthood may also have contributed to healthier individuals and, consequently, improved CAM when compared to those without sports practice in youth. However, it was not possible to clarify this residual confounding in the present study.

Some limitations of the present study should be highlighted. First, the assessment of early sports practice by retrospective questions did not allow for inference about frequency, duration, and intensity, besides being susceptible to memory bias. Second, the lack of assessment of cardiometabolic variables does not allow us to rule out a residual confounding. The small sample size should also be considered as a limitation when compared to other studies [1, 4]. Otherwise, the random sampling process and the objective assessment of current physical activity were strengths of the study. Future research is needed to analyze the role of cardiometabolic parameters, as well as low-grade inflammation, in this relationship,.

## Conclusion

In conclusion, early sports practice was associated with better CAM in adulthood regardless of the current physical activity level. This finding reinforces the need to promote sports practice engagement from early ages, in order to minimize cardiovascular risk in further life stages.

## Data Availability

Data from this study are available from the corresponding author on reasonable request.

## Abbreviations

CAM: Cardiac Autonomic Modulation
PA: Physical Activity
HRV: Heart Rate Variability
MVPA: Moderate to vigorous physical activity
ABEP: Brazilian Market Research Association
